# Viral reactivations following hematopoietic stem cell transplantation in pediatric patients – A single center 11-year analysis

**DOI:** 10.1371/journal.pone.0228451

**Published:** 2020-02-04

**Authors:** Franziska Düver, Benedikt Weißbrich, Matthias Eyrich, Matthias Wölfl, Paul G. Schlegel, Verena Wiegering

**Affiliations:** 1 Department of Oncology, Hematology and Stem Cell Transplantation, University Children’s Hospital Würzburg, Würzburg, Germany; 2 Institute for Virology and Immunobiology, University of Würzburg, Würzburg, Germany; Beth Israel Deaconess Medical Center, UNITED STATES

## Abstract

Viral reactivation occurs frequently in the context of immunodeficiency and immunosuppression after allogeneic hematopoietic stem cell transplantation (allo-HSCT) and can cause severe complications. The aim of this single-center retrospective analysis was to characterize viral infections in the first year after HSCT, to investigate risk factors and to study the impact of viral infections on transplantation outcome. This will facilitate the identification of at-risk patients and the development of new preventive strategies. 107 pediatric allo-HSCT from January 2005 through December 2015 were analyzed for infections with Epstein-Barr virus (EBV), cytomegalovirus (CMV), human herpesvirus 6 (HHV-6), adenovirus (ADV), herpes simplex virus (HSV) and varicella zoster virus (VZV). Viral infections were detected after 68.2% of transplantations. The viruses most commonly encountered were HHV-6 (36/107) and EBV (30/107). Severe viral disease was rare (7/107) and none of the patients died as result of viral reactivation. Important risk factors for viral infections were higher age at HSCT, donor type and occurrence of acute graft-versus-host disease (aGvHD). Especially for EBV, transplant from an unrelated donor and in-vivo T-cell depletion (TCD) had a significant effect on infection rates, whereas for CMV the strongest effect was seen by donor and recipient serostatus with recipient seropositivity most predictive for reactivation. The occurrence of severe aGvHD was associated with EBV and ADV infections. For HSV, the recipient serostatus was identified as prognostic factor for HSV infections, while we found higher age at time of HSCT as risk factor for VZV infections. The overall survival of patients with or without viral infections did not differ significantly. Interestingly, when looking at the 85 patients in our cohort who had received an HSCT for a malignant disease, a tendency towards lower relapse rates was seen in patients affected by viral infections (HR 0.51, 95% CI 0.25 – 1.06, p = 0.072). Viral reactivations are common after pediatric allo-HSCT, though severe complications were rare in our collective. Determining risk factors for viral reactivations may help to identify patients in need of intensified monitoring and to individualize preventive strategies.

## Introduction

Allogeneic hematopoietic stem cell transplantation (allo-HSCT) is an established therapy for a variety of malignancies, congenital and acquired hematological diseases as well as immunodeficiencies. Viral infections account for a large part of complications and even fatalities after HSCT [1–3]. While hygienic measures and isolation of the patient protect against some pathogens, a special risk originates from viruses that silently persist within the patient or the transplanted cells. After primary infection, viruses like Epstein-Barr virus (EBV), cytomegalovirus (CMV), human herpesvirus 6 (HHV-6), adenovirus (ADV), herpes simplex virus (HSV) and varicella zoster virus (VZV) can reactivate especially in the context of immunodeficiency and immunosuppression.

Previous reports on risk factors for viral reactivations after HSCT showed variable results, especially for pediatric patients: An age-dependency, particularly for VZV reactivation in children, was shown by Vermont et al. [4], but the literature available is inconclusive for other members of the herpesvirus family. In patients receiving a human leukocyte antigen (HLA) mismatched or unrelated transplant, viral infection rates tend to be higher than in matched related donors (MRD) [5]. The introduction of haploidentical HSCT made transplants for children without a matching donor possible, but also rendered extensive T-cell depletion (TCD) necessary. The reduced amount of mature immunocompetent cells has been generally associated with a decreased protection against viral infections, especially in adult patients [6].

Graft-versus-host disease (GvHD), one of the main complications after HSCT, as well as its prophylaxis and treatment put patients at risk of viral reactivations [1, 3, 7, 8]. The therapy of simultaneously occurring GvHD (requires increase of immunosuppression) and viral infections (reduction of immunosuppression can be supportive) poses a challenge.

Several studies showed an association between sex mismatch and viral reactivations [9] or the occurrence of GvHD, especially in the combination of a female multiparous donor and a male recipient [10]. Also, some authors suggest a higher risk of infections for ABO blood group antigen incompatible allo-HSCT [11].

To date, most existing studies on viral reactivation focus on an adult collective, although it is well known that pediatric immune recovery differs in terms of velocity and the availability of an intact thymus [12]. There are little data gathered on HHV-6 and ADV reactivations so far, as their significance in the context of HSCT attracted notice only in recent years [13–15]. Additionally, most existing studies only consider one virus, but in most cases, patients are affected by multiple viral reactivations after HSCT.

Therefore, we chose to conduct a 11-year analysis in a pediatric collective of relevant viruses that impose the risk of reactivation: EBV, CMV, HHV-6, ADV, HSV and VZV. First, we characterized incidence and onset of infections with these viruses. Secondly, we studied the association of viral infections (in general and also separately for each virus) with IgG serostatus of donor or recipient, age at time of transplant, donor type, origin of transplant, mismatch of sex or blood type, the use of total body irradiation (TBI), time to engraftment, immune recovery, GvHD and TCD. Thirdly, we evaluated the effect of viral infections on transplantation outcome, relapse rate and overall survival. The obtained data will facilitate the development of individual preventive strategies and the identification of patients in need of intensified monitoring.

## Subjects and methods

### Patients

In this retrospective cohort study, the clinical records and medical charts of children (aged 0–25 years) who underwent allo-HSCT at the University Children’s Hospital Würzburg from January 2005 through December 2015 were analyzed. Two HSCT had to be excluded from the evaluation due to their divergent execution in an emergency setting. If a patient received more than one allogeneic transplant, only the first allo-HSCT was evaluated, with the date of the second transplant defining the end of the follow-up of the first HSCT. This resulted in a total of 107 transplantations available for analysis. The detailed characteristics of the study collective are reported in S1 Table. Patients or legal guardians gave informed consent to scientific analysis and anonymized publication of their medical data in accordance with the Declaration of Helsinki. The ethical committee of the University Hospital Würzburg has determined that for analysis and publication of single center case series this informed consent is sufficient and no specific review of retrospective data analysis projects are required. All data were fully anonymized before statistical analysis.

All patients were treated in conformity with the current guidelines of the GPOH (Gesellschaft für Pädiatrische Onkologie und Hämatologie) and the DAG-KBT (Pädiatrische Arbeitsgemeinschaft für Knochenmarks- und Blutstammzelltransplantation) of that time and conditioned according to disease-specific protocols of the EBMT (European Society for Blood and Marrow Transplantation) working parties.

The main indication for HSCT was leukemia (46.7%). Lymphoma accounted for 10.3% and solid tumors for 11.2% of the transplantations. The remaining 31.8% cover other diseases of the hematopoietic system (e. g. myelodysplastic syndrome, severe aplastic anaemia, beta thalassemia), immunodeficiencies and rare congenital diseases. 11.2% of these other diseases were malignant. Of the 85 patients suffering from a malignant disease, 12.9% showed stable disease (SD, 11/85) at time of transplant. 61.2% were in first complete remission (CR1, 52/85) and 25.9% in the second complete remission (CR2, 22/85). 8.4% of all investigated HSCT were preceded by an autologous transplantation.

The median age on the day of the stem cell infusion was 9.00 years (interquartile range (IQR) = 11.9), with the youngest patient being 2 months and the oldest being 22.2 years old.

Most commonly peripheral blood stem cells (PBSC) were used (80.4%; bone marrow (BM) 18.7%). One patient received a combination of PBSC and BM of the same donor, which we classified as PBSC, and one patient received cord blood (0.9%).

For 28.0% an HLA-identical family donor (MRD) was available. 37.4% underwent transplant from HLA-identical unrelated donors (matched unrelated donor, MUD) and 17.8% from mismatched unrelated donors (MMUD, ≤ 9/10). In another 16.8% the donor was a haploidentical family member.

All patients received a myeloablative conditioning regimen. 38 patients (35.5%) received TBI in the context of conditioning.

In-vitro TCD was conducted in 33.6% of cases. Mostly, CD3^+^/CD19^+^-depletion (16/36, 44.4%) was used. Other depletion schemes included TCRαβ^+^/CD19^+^-depletion (7/36, 19.4%), CD34^+^-selection (1/36, 2.8%) and individual TCD schemes (including only partly T-cell depleted preparations). In-vivo TCD through the application of a total of 30 mg/kg rabbit anti-thymocyte globulin (ATG) shortly before transplant was administered in 66.4% of cases. 23.4% of patients received both, in-vitro and in-vivo TCD. 23.4% of patients did not receive any kind of TCD.

In the majority of cases, patients received cyclosporine A (CSA) as longtime GvHD prophylaxis (82.2%). GvHD prophylaxis with CSA was generally introduced 2 days prior to the transplantation and continued until day 80–120 post-HSCT, depending on signs of GvHD or other individual risk factors. 19.6% received mycophenolate mofetil (MMF), either in addition to CSA or as monotherapy (in extensively T-cell depleted haplo-HSCT). 72.9% of patients received methotrexate (MTX). None of the haploidentical transplants received post-transplant cyclophosphamide.

For the evaluation of acute GvHD (aGvHD), the modified Glucksberg criteria [16] were used and chronic GvHD (cGvHD) was diagnosed based on the criteria demonstrated in Filipovich et al. [17]. In our cohort, 73.8% of the patients (79/107) experienced aGvHD, though mainly a mild form (Grade I: n = 52/79, 65.8%). We saw aGvHD grades II-IV only after 25.2% of HSCT. In 22 cases (20.6%), cGvHD occurred.

Generally, the initial treatment of aGvHD consisted of systemic corticosteroids (2–3 mg/kg prednisone) (57/79; 72.2%). If no response was seen after 7 days or the symptoms increased after 3 days, second line treatment was initiated. This was necessary for 36.7% (29/79) of patients affected by aGvHD and mainly consisted of further immunosuppressants like MMF (24/79) and tacrolimus (4/79) or immunomodulatory agents like etanercept (4/79), tocilizumab (4/79) and others. For 18 patients affected by aGvHD (22.8%), no therapy was initiated.

Therapy of cGvHD consisted of different approaches over the 11 years covered in this study but generally included the use of local or low-dose systemic corticosteroids for limited cGvHD. Extensive cGvHD was mainly treated with high-dose systemic steroids often in combination with other immunosuppressants. Salvage therapy of cGvHD consisted of individual regimens including drugs like CSA, MMF, systemic steroids, MTX, everolimus, etanercept, phototherapy and extracorporeal photopheresis among others.

Engraftment was defined as the first of three consecutive days with a leukocyte count above 1000/μl in peripheral blood samples.

A median observational period of 3.8 years (1393 days; range 22 – 4304 days) was achieved. Short follow-ups were mainly due to early death. For the event-free survival, events were defined as either death or the recurrence of malignant disease, whichever occurred first. If no event occurred until the end of the observation period, the data were censored.

### Monitoring and treatment of viral reactivations

Data on viral infections were obtained from the database of the Institute of Virology and Immunobiology, University of Würzburg. The serostatus for EBV, CMV, HHV-6, HSV and VZV had routinely been acquired at diagnosis of the underlying disease and shortly pre-transplant. Monitoring of viral infections was performed with routine diagnostic polymerase chain reaction (PCR) assays of serum or plasma samples. Qualitative PCR with a threshold of approximately 200 copies were conducted for all examinations of viral infections. Assays were performed as clinical routine in the following intervals: once or twice a week during the first 40 days, weekly from day 40 to 60 and from day 60 on every second week for EBV, CMV, HHV-6 and ADV until cessation of immunosuppression, but at least until day 100. For haploidentical transplantations, the biweekly screening was further continued until CD3^+^ counts were higher than 500/μl. Afterwards, PCR testing was reduced to EBV and CMV only. The screening was intensified and extended when patients showed clinical signs of infections. One or more positive results in the PCR screening were defined as infection; no unambiguous discrimination between reactivation and reinfection could be made in our study setting. HSV and VZV infections were diagnosed by their typical appearance of cutaneous vesical eruptions or at least one positive PCR result of blood samples, vesical fluid or samples obtained from the respiratory tract. Viral disease was defined as proven organ manifestation of one of the evaluated viruses. Data on viral infections until one year after HSCT were included in this study.

All patients received aciclovir at prophylactic doses (20–40 mg/kg/d, preferably enterally) post-HSCT until 4 weeks after cessation of immunosuppression and until they reached a CD4^+^ count > 250/μl. For T-cell depleted HSCT, the antiviral prophylaxis was continued until successful B- and T-cell reconstitution. The medication was paused if the application of another antiviral agent was necessary. Positive EBV levels were only treated with rituximab when accompanied by clinical signs of post-transplant lymphoproliferative disease (PTLD). There was no specific CMV-prophylaxis for CMV seronegative recipients of a CMV-positive transplant. A preemptive CMV therapy was initiated when one blood sample was positive for CMV in the PCR. In our collective mostly foscarnet was used due to its concomitant effectiveness against HSV and VZV. Symptomatic infections that were most likely attributed to HHV-6 were treated with either foscarnet or cidofovir. For ADV, preemptive therapy was considered in highly T-cell depleted patients, otherwise, therapy with cidofovir was initiated when symptoms were most likely attributed to ADV. Aciclovir in therapeutic doses (30 mg/kg/d i.v. + topical medication if possible) was applied in HSV and VZV infections.

### Immune recovery

Routinely, FACS (fluorescence activated cell sorting) analyses of peripheral blood for T-, B- and NK-cell subpopulations were conducted after HSCT. The schedule for the assessment was once a week for day 7–30, every second week until day 60, every fourth week until day 200 and from then on once a year. To assess the speed and quality of immune recovery, three thresholds were defined: > 500 CD3^+^-cells/μl, > 1000 CD3^+^-cells/μl and > 10% CD4^+^CD45RA^+^. The threshold was rated as reached, when 3 consecutive measurements above these values were documented.

### Statistical analysis

Statistical analyses were performed using *SPSS for Windows Version 25* (IBM SPSS Statistics, Chicago, Illinois, USA). All p-values were two-sided with values of ≤ 0.05 indicating statistical significance. Analyses of categorial variables were performed using χ^2^ test and Fisher’s exact test for binary variables. Mann-Whitney U test was used for the comparison of numerical variables of two independent samples with unequal variances. Cox regression with time dependent covariates was used for survival analysis. A Kaplan-Meier function was used for overall survival estimates.

A binary logistic regression analysis was conducted to determine factors associated with viral infections after HSCT independently. The variables age at HSCT, HLA mismatch, unrelated donor, seropositivity of donor (if available), seropositivity of recipient (if available), aGvHD, cGvHD and in-vitro as well as in-vivo TCD were first tested in a univariate analysis. Variables with an p-value < 0.20 in the univariate analysis were considered for the binary regression analysis. The variable with the highest p-value was additionally excluded in “any infection”, “EBV” and “CMV”, as the small cohort limited the number of variables that could be included in the analysis.

## Results

### Characteristics of viral infections

In our pediatric cohort of 107 cases the intended one-year follow-up of virologic data was achieved in 70.1% (75/107) of HSCT. Earlier termination was mainly due to fatalities (n = 25). The median follow-up was 365 days (range = 22–365 days).

**Fig 1:**
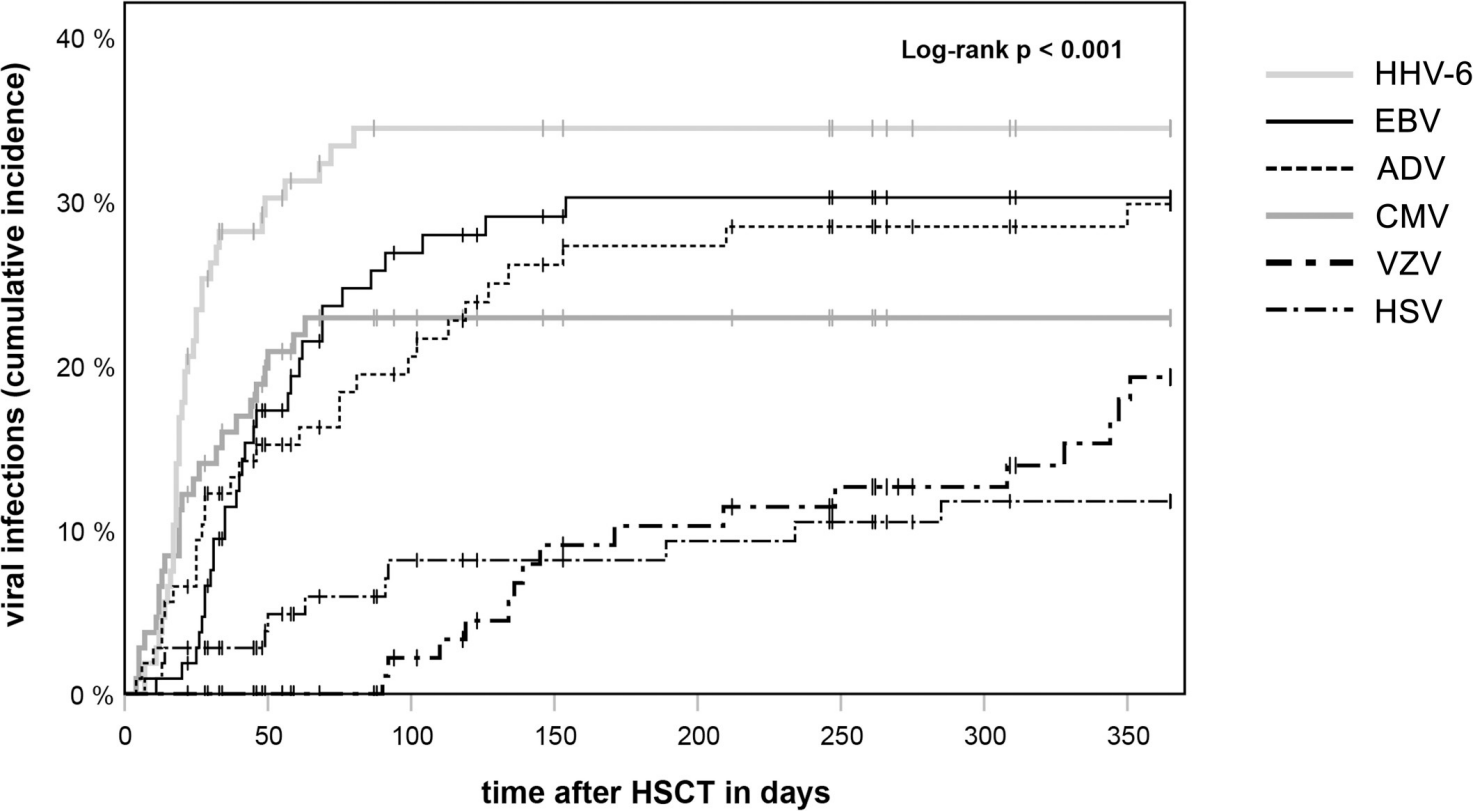
Cumulative incidence of viral infections after HSCT. Time until the first positive testing of infections with Epstein-Barr virus (EBV), cytomegalovirus (CMV), human herpesvirus 6 (HHV-6), adenovirus (ADV), herpes simplex virus (HSV) and varicella zoster virus (VZV) are presented in a Kaplan-Meier graph. Time until first positive testing differed significantly between the tested viruses with a p-value of < 0.001 in the Log-rank test.

Viral infections were detected after 68.2% (73/107) of transplantations. In most cases, patients experienced infections with only one virus (27/73, 37.0%) or two different viruses (26/73, 35.6%) after HSCT. However, the time until the first positive testing significantly varied for the different viruses (p < 0.001; Fig 1). 28.0% of HSCT (n = 30) were affected by EBV infections which occurred after a median of 41.5 days (range = 11–154 days). We observed that HHV-6 was detected most frequently after HSCT (33.6%, n = 36). 14 of these patients were treated with foscarnet, 8 with cidofovir and 2 with both. Infections with HHV-6 occurred very early with a median of 19.5 days (range = 7–80 days) post-transplant, with 58.3% of infections arising in the third or fourth week. Two patients (1.9%) showed constantly high viral load on PCR testing without any symptoms, therefore, we suspected them to have a chromosomally integrated form of HHV-6 infection as reported elsewhere [18–20]. Infections with CMV were detected after 22.4% of HSCT (n = 24) and, like HHV-6, tended to occur early after the transplantation with a median of 19.5 days after HSCT (range = 4–63 days) until the first positive testing. ADV infections made up for the third largest proportion with 29 patients being infected (27.1%). In contrast to the viruses mentioned so far, we saw a wide range of time until detection of ADV (median = 40.0 days, range = 4–350 days). HSV and VZV were observed after 10.3% (n = 11) and 15.0% (n = 16) of HSCT, respectively. VZV infections occurred significantly later than infections with other viruses at a median of 158.0 days post-transplant (range = 90–351 days, p < 0.001), HSV also occurred later (63.0 days, range = 7–285 days), but not significantly compared to other infections.

In every patient affected by CMV or HHV-6, the first positive sample could be documented before day 100. EBV, ADV and HSV infections also mainly emerged before day 100. VZV, in contrast, was never detected in the first 2 months after transplantation, instead VZV reactivations could be detected until the end of our observation period (see Fig 1).

We documented 7 cases of severe viral disease. Two patients suffered from EBV-PTLD and had to be treated with rituximab, another one suffered from EBV tonsillitis and pharyngitis. One patient was diagnosed with HHV-6 pneumonia, another with HHV-6 encephalitis. In one case a patient developed CMV-associated colitis after HSCT and another generalized herpes zoster. Most of these patients had several potential risk factors for viral infections, such as higher age at time of HSCT, MUD, aGvHD or seropositivity of donor or recipient. Importantly, none of the 7 patients with severe viral disease died due to the viral infection.

### Univariate analysis of potential risk factors for viral infections

We studied the association between viral infections and the IgG-serostatus of donor and recipient (D/R; Table 1). The seroprevalence for EBV-IgG was high among stem cell donors (89.2%, 83/93, data missing in 14 cases) compared to recipients before HSCT (48.0%, 48/100, data unavailable for 7 patients). With 42.5% of patients affected, the highest risk of EBV infections was seen in the combination of D+/R-. A tendency for less infections in the combination D+/R+ (20.5%, 8/39) compared to D+/R- (42.5%, 17/40) was seen (p = 0.053). In contrast, transplantation from a seronegative donor never resulted in EBV infections. An EBV positive donor serology was significantly associated with a higher rate of EBV infections (27/83, 32.5%) compared to donors with a negative serology (0/10, 0.0%) (p = 0.032). The difference of EBV infections in patients with negative (17/52, 32.7%) and positive serostatus (11/48, 22.9%) was statistically not significant (p = 0.37).

**Table 1:** Incidence of infections with Epstein-Barr virus (EBV) and cytomegalovirus (CMV), according to the EBV/CMV serostatus of donor (D) and recipient (R). Overall seropositivity for EBV was 89.2% for donors and 48.0% for recipients. For CMV, the overall seropositivity was 41.1% for donors and 24.3% for recipients.

	no EBV infection	EBV infection	no CMV infection	CMV infection
Number of cases (%)	77 (72.0)	30 (28.0)	83 (77.6)	24 (22.4)
**Serostatus donor**, n (%)				
D-	10 (100.0)	0 (0.0)	61 (96.8)	2 (3.2)
D+	56 (67.5)	27 (32.5)	22 (50.0)	22 (50.0)
unknown	11 (78.6)	3 (21.4)	-	-
p-value (χ ^2^)	**0.032**		**< 0.001**	
**Serostatus recipient**, n (%)				
R-	35 (67.3)	17 (32.7)	79 (97.5)	2 (2.5)
R+	37 (77.1)	11 (22.9)	4 (15.4)	22 (84.6)
unknown	5 (71.4)	2 (28.6)	-	-
p-value (χ ^2^)	0.37		**< 0.001**	
**Serostatus donor/recipient**, n (%)				
D or R unknown	15 (75.0)	5 (25.0)	-	-
D-/R-	6 (100.0)	0 (0.0)	60 (100.0)	0 (0.0)
D-/R+	2 (100.0)	0 (0.0)	1 (33.3)	2 (66.7)
D+/R+	31 (79.5)	8 (20.5)	3 (13.0)	20 (87.0)
D+/R-	23 (57.5)	17 (42.5)	19 (90.5)	2 (9.5)

41.1% (44/107) of donors had IgG-antibodies against CMV, in contrast to 24.3% of recipients (26/107) pre-transplant. CMV infections were most frequently detected in CMV seropositive patients receiving a seropositive transplant (20/23, 87.0%), whereas no infections were seen in the combination of both seronegative donor and recipient. A positive CMV serology of the recipient was a significant risk factor for CMV infections (p < 0.001). A higher rate of infections was detected in this collective (22/26, 84.6%) than in patients with a negative CMV serology (2/81, 2.5%). Among patients receiving a CMV seropositive transplant the CMV infection rate was significantly higher (22/44, 50.0%), compared to patients receiving a seronegative transplant (2/63, 3.2%; p < 0.001). The group of CMV seronegative patients receiving a seropositive transplant had a tendency towards more CMV infections (2/21, 9.5%) compared to CMV seronegative patients transplanted with a seronegative preparation (0/60, 0.0%; p = 0.065).

Further data on recipients’ seroprevalence were only available for HHV-6 (91.7%, 88/96, data missing in 11 cases), HSV (36.4%, 36/99, data unavailable in 8 patients) and VZV (78.0%, 78/100, data missing for 7 patients). HSV infections occurred in a higher proportion in patients with a positive HSV serology (10/36, 27.8%) compared to patients with a negative serology (1/63, 1.6%). This difference was significant (p < 0.001). For HHV-6 and VZV, no association of viral infections and the IgG-serostatus of the recipient could be found.

For 33 patients, data on vaccinations were accessible. 10 of them had received one (n = 6) or two (n = 4) doses of VZV vaccine prior to HSCT. 23 patients had received no VZV vaccination of which 5 (21.7%) had an VZV infection after HSCT, while no vaccinated patient had a VZV reactivation.

Patients who suffered from one or more viral infections after HSCT were significantly older (median = 10.1 years) than those without infections (median = 3.4, p-value: < 0.001). When considering the different viruses separately, we found that patients experiencing a VZV infection after HSCT were significantly older than patients who did not (median = 14.6 years vs. 8.2 years, p = 0.004). VZV seropositivity increased significantly with age (p < 0.001). Also, patients experiencing HSV infections were significantly older than patients who did not (median = 14.0 years vs. 8.2 years, p = 0.025). Other viruses did not show significant age-dependency.

Considering donor type, the highest infection rates were recorded in patients receiving a MMUD transplant (16/19, 84.2%) and the lowest for MRD (15/30, 50.0%). In MUD and haploidentical transplantation, 70.0% (28/40) and 77.8% (14/18) of patients experienced infections in our cohort, respectively. This varied when looking at the viruses separately: For EBV, we saw infections mainly in patients receiving a transplant from an unrelated donor (MUD 42.5% (17/40) and MMUD 52.6% (10/19) vs. MRD 6.7% (2/30) and haploidentical HSCT 5.6% (1/18)). This association was significant (p < 0.001). In contrast, CMV infections preferably occurred in HLA-mismatched settings (MMUD 26.3% (5/19) and haploidentical HSCT 38.9% (7/18) vs. MRD 16.7% (5/30) and MUD 17.5% (7/40); p-value = 0.090). HHV-6, ADV, HSV and VZV all had the smallest infection rate for MRD, although a significant association between the type of donor and rate of infections was only discernible for HHV-6. Most HHV-6 infections were seen in haploidentical HSCT.

There was no significant relationship between infections and the stem cell source. In addition, no significant association were found for TBI conditioning or the mismatch of blood type. For sex mismatch, significantly more infections were only found for HSV (17.0% vs. 3.7%, p-value = 0.029).

3/107 transplants (2.8%) failed to engraft. The affected patients died very shortly after HSCT (days 22, 28 and 33) from complications. One of these patients had already been tested positive for EBV and HHV-6, another for CMV, but in none of these patients were viral infections the cause of death. The median time to engraftment was 15.0 days (IQR = 6). There was no significant difference in time to engraftment between patients with (median = 14.0 days) and without infections after HSCT (median = 16.0 days). We registered 3 cases of graft failure after engraftment, mostly in the first month after transplant (days 16, 21 and 86). In one patient the graft failure was preceded by a CMV infection, in another patient by an HHV-6 infection.

The goal of > 500/μl CD3^+^-cells was reached with a median of 133.5 days post-HSCT (IQR = 160) and > 1000/μl CD3^+^-cells were reached with a median of 266.5 days (IQR = 286). Patients with infections after HSCT did not reach the thresholds defined for CD3^+^-cells significantly later than those without infections, however, we found a significant difference for the threshold of > 10% CD4^+^CD45RA^+^-cells (p = 0.014): While patients without viral infections reached the threshold with a median time of 102.0 days (IQR = 114), those with infections reached it at a median time of 154.0 days (IQR = 166). Also, significantly less patients reached the goal of > 10% CD4^+^CD45RA^+^-cells in the group with viral infection (HR 0.51, 95% CI 0.30–0.85, p = 0.010). For the threshold of > 500/μl CD3^+^-cells and > 1000/μl CD3^+^-cells no significant difference could be shown.

Data on GvHD are presented in S2 Table. We found no significant difference of infection rates between patients with and without aGvHD (72.2% vs. 57.1%, p = 0.16). Considering the viruses separately, we found significantly more ADV infections in patients suffering from aGvHD (32.9% vs. 10.7%, p = 0.026). All patients with severe aGvHD (grade III or IV) had at least one viral infection and there were significantly more infections in this group than in patients having no or only mild aGvHD (grade I or II) (100% vs. 63.4%; p = 0.004). Considering this association for the viruses separately, we found that patients with severe aGvHD had a significantly higher incidence of EBV (57.1% vs. 23.7%; p = 0.021) and ADV infections (78.6% vs. 19.4%; p < 0.001) than those suffering from no or only mild aGvHD. There was no significant association of the occurrence of cGvHD and the occurrence of viral infections.

In our pediatric cohort of 107 cases, 33.6% had received an in-vitro T-cell depleted transplant. As presented in Table 2, a significant association of in-vitro TCD and CMV infections after HSCT was seen. In-vivo TCD through the application of ATG was conducted in 66.4% of cases. Significantly more infections with EBV were seen in patients who had received ATG (29/71, 40.8%) compared to patients who did not undergo in-vivo TCD (1/36, 2.8%; p < 0.001). A general tendency towards more infections in the in-vivo depleted group was seen. Of the 7 patients affected by severe viral diseases, 4 (suffering from HHV-6 encephalitis, generalized zoster and two cases with suspected EBV-PTLD) had received in-vitro T-cell depleted preparations. These 4 patients and the patient affected by EBV tonsillitis and pharyngitis had also been treated with ATG.

**Table 2:** Incidence of viral infections for (a) in-vitro T-cell depletion (TCD) and (b) in-vivo TCD (anti-thymocyte globulin).

(a)			
infection rates	no in-vitro TCD, n = 71	in-vitro TCD, n = 36	p-value (Fisher)
any infection, n (%)	45 (63.4)	28 (77.8)	0.19
EBV infection, n (%)	22 (31.0)	8 (22.2)	0.37
CMV infection, n (%)	11 (15.5)	13 (36.1)	**0.026**
HHV-6 infection, n (%)	19 (26.8)	17 (47.2)	0.051
ADV infection, n (%)	19 (26.8)	10 (27.8)	> 0.999
HSV infection, n (%)	6 (8.5)	5 (13.9)	0.50
VZV infection, n (%)	11 (15.5)	5 (13.9)	> 0.999
(b)			
infection rates	no in-vivo TCD, n = 36	in-vivo TCD, n = 71	p-value (Fisher)
any infection, n (%)	20 (55.6)	53 (74.6)	0.051
EBV infection, n (%)	1 (2.8)	29 (40.8)	**< 0.001**
CMV infection, n (%)	4 (11.1)	20 (28.2)	0.052
HHV-6 infection, n (%)	12 (33.3)	24 (33.8)	> 0.999
ADV infection, n (%)	7 (19.4)	22 (31.0)	0.25
HSV infection, n (%)	3 (8.3)	8 (11.3)	0.75
VZV infection, n (%)	4 (11.1)	12 (16.9)	0.57

### Multivariate analysis of potential risk factors for viral infections

In the multivariate analysis (Table 3), higher age at HSCT, HLA mismatch and aGvHD were identified as independent risk factors for viral infections after HSCT. Patients who received an HLA mismatched graft were 3.92 times (95% CI: 1.24 – 12.4; p = 0.020) more likely to develop viral infections after HSCT. Considering EBV, transplants from an unrelated donor and in-vivo TCD were associated significantly with a higher infection rate, whereas higher age at HSCT was not significant in the multivariate analysis. CMV seropositive recipients had the highest odds for CMV infection (OR 100.31; 95% CI: 15.94 – 631.38; p < 0.001), but a significant effect of CMV seropositivity of the donor on infection rates was also observed (OR 11.59; 95% CI: 1.22 – 109.95; p = 0.033). Patients with positive HSV serostatus were 20.93 times (95% CI: 2.51 – 174.49; p = 0.005) more likely to develop HSV infections than those with negative HSV serostatus. For HHV-6 and ADV no significant variable was identified in the multivariate analysis. VZV infections were not tested in a binary logistic regression analysis as only one variable with a p-value < 0.20 in the univariate analysis had been identified.

**Table 3:** Binary logistic regression analysis of risk factors for viral infections after HSCT.

	odds ratio	95% confidence interval	p-value
**any infection**			
age at HSCT	1.24	1.12–1.37	**< 0.001**
HLA mismatch	3.92	1.24–12.40	**0.020**
unrelated donor	1.16	0.27–5.02	0.85
aGvHD	3.39	1.08–10.63	**0.036**
in-vivo TCD	2.81	0.62–12.71	0.18
**EBV**			
age at HSCT	1.08	1.00–1.17	0.057
unrelated donor	5.05	1.24–20.63	**0.024**
in-vivo TCD	10.68	1.15–98.86	**0.037**
**CMV**			
seropositivity donor	11.59	1.22–109.95	**0.033**
seropositivity recipient	100.31	15.94–631.38	**< 0.001**
in-vitro TCD	2.54	0.38–16.85	0.34
**HHV-6**			
age at HSCT	1.07	0.99–1.14	0.078
HLA mismatch	1.54	0.54–4.35	0.42
aGvHD	2.43	0.85–6.95	0.099
in-vitro TCD	2.08	0.73–5.93	0.17
**ADV**			
aGvHD	3.59	0.97–13.33	0.056
cGvHD	1.73	0.63–4.76	0.29
**HSV**			
age at HSCT	1.11	0.97–1.28	0.14
seropositivity recipient	20.93	2.51–174.49	**0.005**

### Effects of viral infections

Overall mortality was 28.0% (30/107) in our cohort, of which 9 fatalities (9.3%) were transplant-related (TRM = transplant-related mortality) and 19 (17.8%) were categorized as dead of disease (DOD). One patient died from a parainfluenza infection (0.9%). DOD was the main cause of death in patients with and without infections after HSCT. There was no significant association of viral infections and DOD (11/73, 23.5% vs. 8/34, 15.1%; p-value = 0.29) or TRM (8/73, 11.0% vs. 1/34, 2.9%; p–value = 0.27).

The 1-year- and 2-year-survival for our collective was 80.6% and 76.5% respectively. The overall survival of patients with or without infections after HSCT did not differ significantly (HR 1.14, 95% CI 0.52 – 2.47, p = 0.75).

Of the 85 patients who had been transplanted because of a malignant disease, 31 (36.5%) relapsed or experienced progress of the primary disease. In the cox regression analysis, a tendency towards more relapses and progresses of the primary disease were found in patients without viral infections compared to those with infections (HR 0.51, 95% CI 0.25 – 1.06, p = 0.072). The small number of patients in the subgroups precluded a virus-specific analysis.

## Discussion

Reactivation of viral infections with EBV, CMV, HHV-6, ADV, HSV and VZV after HSCT is common, as we could confirm in this single-center retrospective study. The aim of our report was to assess risk factors for and impacts of infections with these viruses in pediatric patients.

For EBV, CMV, HHV-6 and ADV we showed a temporal concurrence with the time of peak immunosuppression after HSCT, with all patients affected by CMV or HHV-6 infections developing viremia in the first 100 days post-transplant. Additionally, the majority of EBV and ADV infections were first detected in this period.

The incidence of EBV infection in our collective was comparable to other pediatric studies reporting infections after 22 – 32% of HSCT [21, 22]. Little data are available regarding the donor and recipient serostatus for EBV in pediatric patients. For EBV we showed the highest infection rates in D+/R- and no infections in patients receiving an EBV-negative transplant. Meijer et al. suggest, that EBV infection after HSCT frequently results from reinfection with an exogenous EBV strain instead of a true reactivation, but do not investigate whether this strain derives from the donor [23]. Interestingly, there seems to be a protective effect of EBV-seropositivity in patients receiving cells of an EBV-seropositive donor, as we found a tendency towards less infections in the combination D+/R+ compared to D+/R-. A similar protective effect was demonstrated by Bordon et al. [21].

The incidence of CMV infections in our study was similar to the 22 – 24% reported in other pediatric studies [1, 7, 24]. Consistent with these studies, we were able to show that recipient seropositivity had a significant effect on the incidence of CMV infection. Because of the significantly lower rate of CMV infections in seronegative patients, our data suggest that the reactivation of latent CMV in the host is the most frequent cause of CMV infection after HSCT. Furthermore, many studies were able to show advantages of a CMV seronegative donor for a seronegative recipient and this is also recommended uniformly in current guidelines [8, 25]. Our data support this recommendation, as we saw a trend towards less infections in D-/R- compared to D+/R-. Especially in pediatric collectives, the rate of CMV infections in CMV seropositive recipients varies. While Qayed et al. and Yoon et al. showed the highest incidence for CMV infections in D+/R+ [7, 26], other authors found the highest incidence in D-/R+ [24, 27, 28]. The reported rates of CMV infection in these pediatric studies varied from 17 to 71% for D-/R+, which is in line with our study, and 24 to 57% for D+/R+, which is lower than in our collective.

HHV-6 viremia is detected frequently after pediatric HSCT, with more than a third of transplantations being affected [13, 29]. In two patients (1.9%), we suspected a chromosomal integrated form of HHV-6 due to constantly high viral load and missing clinical symptoms. This phenomenon has been reported with an incidence of 1 – 4% for the total population in previous studies [18–20, 30]. Though very rare, it is important to keep this in mind to avoid unnecessary antiviral treatment of these patients, for example the escalation of potentially toxic antiviral therapy when no response in viral load is seen. It is still unclear to which extent chromosomally integrated HHV-6 can be activated after HSCT and induce pathogenic effects [30].

The risk of ADV infections is elevated in pediatric patients and an increase of infection rates has been reported over the last decades [31]. The incidence of ADV infections in our study is in line with 25–50.4% determined in other studies [32, 33]. The infections are mainly due to reactivation of asymptomatic ADV persistence [33].

As expected, VZV occurred significantly later than infections with other viruses, which is very likely due to the use of an extended prophylaxis as seen in other studies using aciclovir [34, 35, 36, 37]. HSV is classically seen as a virus reactivating very early, especially in the first 4 weeks after HSCT [35, 38, 39, 40]. However, in our study cohort aciclovir prophylaxis was routinely used and HSV was not under active surveillance. Here, in 8/11 cases, HSV reactivation was seen later than one month after HSCT. Additionally, we registered very low rates of HSV and VZV reactivations with 10.3% and 15.0% respectively compared to studies without routine aciclovir prophylaxis [4, 39, 41, 36]. However, these findings are in line with the few data existing on pediatric cohorts receiving aciclovir prophylaxis: Maltezou et al. for example detected an incidence of 14% for HSV infections in the first two years after HSCT [42] while Aytaҫ et al. and Han et al. recorded rates of 13.6% and 11.2% respectively for VZV reactivations in the first year after HSCT [34, 37]. For HSV, significantly more infections occurred in seropositive than in seronegative recipients; this suggests that HSV infections post-HSCT are mainly due to reactivation of HSV in seropositive patients.

We saw less VZV infections in patients vaccinated prior to HSCT, but as data on VZV vaccination were limited in our study, this association was not significant. Further research into VZV vaccination is needed to evaluate the effect on VZV reactivation after HSCT, though some reviews already suggest vaccination of VZV seronegative HSCT recipients at least 4 weeks before start of conditioning [43, 44]. A study on a German pediatric cohort was able to show a decline of VZV infections in oncology patients which might partly be attributed to the universal recommendation for varicella vaccination that was introduced in Germany in 2004 [45].

The occurrence of severe aGvHD was associated with increased rates of EBV and ADV infections. As viral infections and aGvHD occur at the same early post-transplant period, it is difficult to make a statement about causality. A different study design will be necessary to differentiate whether GvHD increases rates of viral infections or whether viral infections increase the risk of GvHD.

Patients suffering from viral reactivation were significantly older than those who were not affected, which could be due to seroprevalence increasing with age, but also due to a slower thymic recovery in older children [46, 47]. In line with our findings, Berman et al. were able to indicate an age > 10 years as a risk factor for VZV infections [41]. Vermont et al. also showed that pediatric allo-HSCT patients suffering from VZV infections were significantly older than those who did not have VZV infections after transplantation [4]. Additionally, we were able to show a similar age-dependency for HSV, but not for the other viruses included in this study.

As expected, the highest infection rates were seen in patients who underwent HSCT from a MMUD. In patients receiving an HLA-mismatched or unrelated transplant, viral infection rates tend to be higher than in MRD, probably due to the necessity of an intensified GvHD prophylaxis with immunosuppressive agents or the increased use of TCD. Additionally, the occurrence of GvHD itself can delay immune reconstitution and therapeutic measurements may include an augmented immunosuppression. For EBV, the lowest risk of infection was seen in haploidentical HSCT, which is probably due to B-cell depletion in these patients. The highest risk of EBV infection was seen in patients receiving an unrelated transplant, as described by Bordon et al. [21]. In contrast, most CMV infections were documented in patients who underwent HSCT from a mismatched donor. The increased use of in-vitro TCD in the setting of mismatched transplantation, compared to unrelated donors, might explain this observation, as significantly more CMV infections were seen in patients receiving an in-vitro T-cell depleted graft.

Except for CMV, a significant association of in-vitro T-cell depleted HSCT with increased rates of viral infections could not be found. For in-vivo TCD, significantly more infections were only observed for EBV. Some tendencies towards more infections in the depleted group were discernible and most patients with severe viral disease had received TCD, but the effect was much milder than expected. This might be due to the heterogeneity of the cohort with many different forms of TCD and the low number of subjects in general. Also, patients transplanted with T-cell depleted graft might have received a longer aciclovir prophylaxis due to a slower immune reconstitution. The negative effect of TCD on infection rates after HSCT was mainly shown for adult collectives and attributed to a slower immune reconstitution. It is conceivable that the impact might be smaller in children due to their generally different and faster immune reconstitution.

Stem cell source, TBI and mismatch of blood type had no significant effect on infection rates.

In one patient failed engraftment was preceded by a CMV infection, in another patient by an HHV-6 infection, but we assume that these infections were concomitant and not cause of rejection. No difference in the velocity of immune reconstitution for CD3^+^-cells was seen when comparing patients with and without viral infections after HSCT, though the goal of > 10% CD4^+^CD45RA^+^-cells was reached faster in patients not affected by viral infections. This might indicate that the reconstitution of a more diverse immune system reduces the vulnerability to viral infections.

Interestingly, when looking at the 85 patients in our cohort who had received an HSCT for a malignant disease, a tendency towards a lower relapse risk was seen in patients affected by viral infections. Several studies showed similar results for CMV, especially in acute myeloid leukemia (AML) patients [48–50], Auger et al. for EBV and Nordlander et al. for HSV infections [51, 52]. The underlying mechanism of this relapse reduction remains unknown. It is hypothesized, that this observation might be accounted for by virally triggered expression of peptides by AML blasts which can serve as target for immune cells and thereby increase the graft-versus-leukemia (GvL) effect [49].

There are little data on viral infections after HSCT in pediatric patients. The consistent development of new conditioning and prophylaxis schemes as well as new forms of transplantations make new studies necessary. We chose to observe several viruses in the same collective to better represent the fact that most patients are affected by multiple viral infections after HSCT.

The retrospective character of this study with data of a single institution limits the transferability of our findings to other collectives. To verify our findings, a prospective multicenter study should be conducted. An acquisition of quantitative data on viral load might provide additional insights. The use of a uniform detection method in the context of a prospective analysis would make the acquired data more reliable. Furthermore, the impact of routine VZV vaccination on the development of VZV infections after HSCT needs to be investigated more extensively. In order to explore the effect of TCD on viral reactivation in children in greater detail, larger prospective studies with homogenous cohorts should be performed.

In conclusion, viral infections are very common after allo-HSCT in children. However, our data suggest that severe viral disease and death due to viral reactivation are rare. Age at time of transplantation, the occurrence of aGvHD and donor type were the most important risk factors for viral infections after HSCT in general. Especially for EBV, transplant from an unrelated donor and in-vivo TCD had a significant effect on infection rates, whereas for CMV the strongest effect was seen by donor and recipient serostatus with recipient seropositivity most predictive for reactivation. The occurrence of severe aGvHD was associated with EBV and ADV infections. In HSV infections, the recipient serostatus was identified as prognostic factor for HSV infections, while we found higher age at time of HSCT as risk factor in VZV infections. These evaluated risk factors allow further tailoring of prophylaxis strategies for children, who are at the highest risk of viral infections.

## Supporting information

S1 Table Patient demographics and transplantation characteristics. SD indicates stable disease; CR1, first complete remission; CR2, second complete remission; MRD, matched related donor; MUD, matched unrelated donor; MMUD, mismatched unrelated donor; PBSC, peripheral blood stem cells; BM, bone marrow; CB, cord blood; TBI, total body irradiation; ATG, anti-thymocyte globulin; GvHD, graft-versus-host disease; MTX, methotrexate; OKT-3, Muromonab CD3; CSA, cyclosporine A; MMF, mycophenolate mofetil; IQR, interquartile range; aGvHD, acute graft-versus-host disease; cGvHD, chronic graft-versus-host disease; TRM, transplant-related mortality; DOD, dead of disease. *Other schemes include individual TCD and TCD conducted only on a part of the graft.
(DOCX)

S2 Table Incidence of viral infections in patients with aGvHD, high-grade aGvHD and cGvHD after HSCT. (DOCX)
