# Supplementary material for: Viral reactivations following hematopoietic stem cell transplantation in pediatric patients – A single center 11-year analysis
Source: PLoS One. 2020 Feb 4;15(2):e0228451. doi: 10.1371/journal.pone.0228451 (PMC6999888; doi:10.1371/journal.pone.0228451)
Supplement: S1 Table — SD indicates stable disease; CR1, first complete remission; CR2, second complete remission; MRD, matched related donor; MUD, matched unrelated donor; MMUD, mismatched unrelated donor; PBSC, peripheral blood stem cells; BM, bone marrow; CB, cord blood; TBI, total body irradiation; ATG, anti-thymocyte globulin; GvHD, graft-versus-host disease; MTX, methotrexate; OKT-3, Muromonab CD3; CSA, cyclosporine A; MMF, mycophenolate mofetil; IQR, interquartile range; aGvHD, acute graft-versus-host disease; cGvHD, chronic graft-versus-host disease; TRM, transplant-related mortality; DOD, dead of disease. *Other schemes include individual TCD and TCD conducted only on a part of the graft. (DOCX) [file pone.0228451.s001.docx]

| characteristic |  | |
| --- | --- | --- |
| gender, n (%) |  |  |
| male | 60 | (56.1) |
| female | 47 | (43.9) |
| median age in years (range) | 9.0 | (0.2 - 22.2) |
| diagnosis, n (%) |  |  |
| leukemia | 50 | (46.7) |
| lymphoma | 11 | (10.3) |
| solid tumor | 12 | (11.2) |
| others benign | 22 | (20.6) |
| others malign | 12 | (11.2) |
| malignant disease, n (%) | 85 | (79.4) |
| Status at the time of transplant |  |  |
| SD | 11/85 | (12.9) |
| CR1 | 52/85 | (61.2) |
| CR2 | 22/85 | (25.9) |
| donor type, n (%) |  |  |
| MRD | 30 | (28.0) |
| MUD | 40 | (37.4) |
| MMUD | 19 | (17.8) |
| haploidentical | 18 | (16.8) |
| stem cell source, n (%) |  |  |
| PBSC | 86 | (80.4) |
| BM | 20 | (18.7) |
| CB | 1 | (0.9) |
| TBI conditioning, n (%) | 38 | (35.5) |
| no T-cell depletion, n (%) | 25 | (23.4) |
| only in-vitro T-cell depletion, n (%) |  |  |
| CD34⁺-selection | 1 | (0.9) |
| CD3⁺/CD19⁺-depletion | 8 | (7.5) |
| TCRαβ⁺/CD19⁺-depletion | 0 | (0.0) |
| other schemes* | 2 | (1.9) |
| only in-vivo T-cell depletion (ATG), n (%) | 46 | (43.0) |
| in-vivo and in-vitro TCD, n (%) |  |  |
| CD34⁺-selection | 0 | (0.0) |
| CD3⁺/CD19⁺-depletion | 8 | (7.5) |
| TCRαβ⁺/CD19⁺-depletion | 7 | (6.5) |
| other schemes* | 10 | (9.3) |
| mismatch sex, n (%) | 53 | (49.5) |
| mismatch blood type, n (%) |  |  |
| no | 52 | (48.6) |
| yes | 54 | (50.5) |
| indeterminable | 1 | (0.9) |
| GvHD prophylaxis, n (%) |  |  |
| only CSA | 75 | (70.1) |
| only MMF | 9 | (8.4) |
| CSA + MMF | 9 | (8.4) |
| others | 14 | (13.1) |
| median follow-up in days (IQR) | 1393.0 | (1860) |
| GvHD, n (%) |  |  |
| acute | 79 | (73.8) |
| chronic | 22 | (20.6) |
| aGvHD localization, n (%) |  |  |
| skin | 77/79 | (97.5) |
| liver | 12/79 | (15.2) |
| gastrointestinal | 13/79 | (16.5) |
| aGvHD grade, n (%) |  |  |
| I | 52/79 | (65.8) |
| II | 13/79 | (16.5) |
| III | 11/79 | (13.9) |
| IV | 3/79 | (3.8) |
| aGvHD therapy, n (%) |  |  |
| none | 18/79 | (22.8) |
| steroids | 57/79 | (72.4) |
| additional second line treatment | 29/79 | (36.7) |
| cGvHD localization, n (%) |  |  |
| skin | 14/22 | (63.6) |
| liver | 10/22 | (45.5) |
| gastrointestinal | 7/22 | (31.8) |
| lung | 5/22 | (22.7) |
| joints/rheumatic | 4/22 | (18.2) |
| cGvHD score, n (%) |  |  |
| 1 | 6/22 | (27.3) |
| 2 | 7/22 | (31.8) |
| 3 | 9/22 | (40.9) |
| outcome, n (%) |  |  |
| alive | 72 | (67.3) |
| TRM | 11 | (10.3) |
| DOD | 22 | (20.6) |
| other cause of death | 2 | (1.8) |
| malignant disease, n (%) | 85 | (79.4) |
| relapse/progress after HSCT, n (%) | 31/85 | (36.5) |
