# Supplementary material for: Viral reactivations following hematopoietic stem cell transplantation in pediatric patients – A single center 11-year analysis
Source: PLoS One. 2020 Feb 4;15(2):e0228451. doi: 10.1371/journal.pone.0228451 (PMC6999888; doi:10.1371/journal.pone.0228451)
Supplement: S2 Table — (DOCX) [file pone.0228451.s002.docx]

|  | **any infection** | | **EBV** | | **CMV** | | **HHV-6** | | **ADV** | | **HSV** | | **VZV** | |
| --- | --- | --- | --- | --- | --- | --- | --- | --- | --- | --- | --- | --- | --- | --- |
|  | – | + | – | + | – | + | – | + | – | + | – | + | – | + |
| aGvHD, n |  |  |  |  |  |  |  |  |  |  |  |  |  |  |
| no | 12 | 16 | 23 | 5 | 22 | 6 | 22 | 6 | 25 | 3 | 26 | 2 | 23 | 5 |
| yes | 22 | 57 | 54 | 25 | 61 | 18 | 49 | 30 | 53 | 26 | 70 | 9 | 68 | 11 |
| p-value (χ²) | 0.16 | | 0.22 | | > 0.99 | | 0.16 | | **0.026** | | 0.72 | | 0.76 | |
| aGvHD, n |  |  |  |  |  |  |  |  |  |  |  |  |  |  |
| none, I-II | 34 | 59 | 71 | 22 | 74 | 19 | 64 | 29 | 75 | 18 | 82 | 11 | 80 | 13 |
| III-IV | 0 | 14 | 6 | 8 | 9 | 5 | 7 | 7 | 3 | 11 | 14 | 0 | 11 | 3 |
| p-value (χ²) | **0.004** | | **0.021** | | 0.30 | | 0.23 | | **< 0.001** | | 0.35 | | 0.44 | |
| cGvHD, n |  |  |  |  |  |  |  |  |  |  |  |  |  |  |
| no | 28 | 57 | 60 | 25 | 63 | 22 | 54 | 31 | 65 | 20 | 77 | 8 | 74 | 11 |
| yes | 6 | 16 | 17 | 5 | 20 | 2 | 17 | 5 | 13 | 9 | 19 | 3 | 17 | 5 |
| p-value (χ²) | 0.80 | | 0.61 | | 0.15 | | 0.31 | | 0.11 | | 0.69 | | 0.31 | |
